# Extraction, Identification, and Antioxidant Activity of Flavonoids from *Hylotelephium spectabile* (Boreau) H. Ohba

**DOI:** 10.3390/foods13172652

**Published:** 2024-08-23

**Authors:** Na Li, Xiao Wu, Qin Yin, Zeng Dong, Lele Zheng, Yihui Qian, Yulu Sun, Ziping Chen, Kefeng Zhai

**Affiliations:** 1 School of Biological and Food Engineering, Suzhou University, Suzhou 234000, China; li60989211@126.com (N.L.); szxywuxiao@163.com (X.W.); qinyin@ahszu.edu.cn (Q.Y.); dongzeng@ahszu.edu.cn (Z.D.); zhengll2024@yeah.net (L.Z.); 18856298036@163.com (Y.Q.); 19855961914@163.com (Y.S.); 2 Anhui Promotion Center for Technology Achievements Transfer, Anhui Academy of Science and Technology, Hefei 230031, China; 3 Engineering Research Center for Development and High Value Utilization of Genuine Medicinal Materials in North Anhui Province, Suzhou 234000, China

**Keywords:** *Hylotelephium spectabile* (Boreau) H. Ohba leaves total flavonoids, response surface methodology, macroporous adsorption resin separation and purification, HPLC-MS, antioxidant activity

## Abstract

The extraction of total flavonoids from *Hylotelephium spectabile* (Boreau) H. Ohba (*H. spectabile*) leaves was studied through the use of a double enzyme-assisted ultrasonic method, and the extraction process was optimized using the Box–Behnken design. Eight different macroporous resins were screened for purification in single-factorial experiments, and the flavonoid compounds in the extract of *H. spectabile* leaves were identified using HPLC-MS. Through the evaluation of the total reducing capacity and capacity for reducing 1,1-diphenyl-2-trinitrophenylhydrazine (DPPH), hydroxyl radicals (·OH), and 2,2’-biazobis(3-ethylbenzothiazoline-6-sulfonic acid) diammonium salt (ABTS), the in vitro antioxidant activities of the crude extracts of the total flavonoids and purified total flavonoids of *H. spectabile* leaves were investigated. The results showed that the most efficient conditions for flavonoid extraction were an ultrasonic extraction time of 60 min, an ethanol concentration of 35%, a liquid-to-material ratio of 20:1 mL/g, and an amount of enzyme (cellulose/pectinase = 1:1) of 1.5%, forming *H. spectabile* powder. Under these conditions, the total flavonoid extraction rate in the *H. spectabile* leaf extract was 4.22%. AB-8 resin showed superior performance in terms of purification, and the optimal adsorption and desorption times were 1.5 h and 3 h, respectively. The recommended parameters for purification included a liquid volume of 5.5 BV, a flow rate of 1.2 BV/min, a pH of 5, and a concentration of 0.8 mg/mL. The observed order for reducing capacity was ascorbic acid (VC) > rutin > purified total flavonoids > crude extract of total flavonoids. The purified total flavonoid extract from *H. spectabile* showed a good scavenging ability against DPPH, ·OH, and ABTS^·+^, suggesting strong antioxidant activity. Therefore, this study can serve as technical support and reference data for the further development and utilization of *H. spectabile* resources.

## 1. Introduction

*H. spectabile*, also known as scorpion grass, is a species of dicotyledonous plant in the *Sedum* family that can be used to reduce temperature, detoxify the body, dispel wind, increase water retention, promote blood circulation to the skin, relieve pain, and aid in hemostasis [1]. It is complex and contains a variety of biofunctional substances, such as alkaloids, steroids, tannins, flavonoids, and their derivatives. Flavonoids mainly refer to the basic parent nucleus of 2-phenylchromogenic ketone compounds [2], which are a large class of secondary metabolites formed through long-term natural selection in plants. They exhibit activity against several conditions, such as diabetes, inflammation, and oxidative processes [3]. They also exhibit various pharmacological effects, including anti-tumor, antibacterial, antiviral, anti-cancer, anti-aging, anti-depression, anti-cardiovascular, and cerebrovascular diseases properties, as well as the regulation of blood lipids and the improvement in immunity [4,5,6]. With the growing global demand for plant extract products, the extraction of natural flavonoids from plants with high purity, activity, safety, and non-toxicity has become a hotspot for natural drug research and development in recent years [7]. With the development of modern extraction and separation technology, many new flavonoids have been discovered, and many flavonoid-containing medicines and health foods have been developed, with great economic and social benefits [8]. The rate at which total flavonoids are extracted from *H. spectabile* leaves also varies according to the extraction conditions. Enzymolysis and ultrasonic extraction methods have been combined, and organic solvents with high extraction rates have been used to extract the active compounds [9]. Box–Behnken analysis has been used to establish a model to determine the appropriate extraction-process conditions using enzyme-assisted ultrasound with the highest extraction yields [10]. However, the total flavone extract contains a large number of impurities, such as pigments, cellulose, polysaccharides, and proteins, resulting in a very low purity of the total flavonoids. This impurity issue complicates further experiments, necessitating purification treatment [11].

Macroporous resin is a polymer in nature and is widely used in flavonoid separation and purification. In addition, macroporous resin has a good separation effect on polyphenols and flavonoids [12]. The flavonoid extract was purified by macroporous resin, and its antioxidant activity was tested based on the improved extraction rate and purity of total flavonoids. Its content was determined by the HPLC-MS in *H. spectabile* [13]. Therefore, the extraction and purification of flavonoids with high biological activity from natural plants and the further development of drugs and functional foods will certainly promote the development of China’s medicine and food industry and are of great significance for the promotion of human health.

In this study, the extraction and purification process of total flavonoids from *H. spectabile* was optimized, and the main flavonoids were detected by HPLC-MS. The antioxidant capacity was also further clarified by in vitro antioxidant experiments, which provided theoretical and technical support for the further development and utilization of the total flavonoids from *H. spectabile.*

## 2. Materials and Methods

### 2.1. Materials and Reagents

*H. spectabile* leaves were collected according to a completely randomized design with three replicates from the plant’s growing sites in Jiangsu *H. spectabile* Horticultural Base, Jiangsu Province. Specifically, three samples were collected from each plant, and they were mixed before the samples were labeled.

Rutin standard, pectinase (50 μ·mg ^−1^), and cellulose (50 μ·mg ^−1^) were purchased from Sinopharm Group Chemical Reagent Co., Ltd. (Wuhan, China). D101, AB-8, HPD-826, NKA-9, HPD-826, ADS-17, HPD-600, and S-8 macroporous resins with different polarity were all purchased from Beijing Solaibo Technology Co., Ltd. (Beijing, China). Ascorbic acid (VC) was purchased from Sinopharm Group Chemical Reagent Co., Ltd. (Wuhan, China). DPPH (1, 1-diphenyl-2-trinitrophenylhydrazine) was purchased from Shanghai Yuanye Biotechnology Co., Ltd. (Shanghai, China). Tris was purchased from Shanghai Qiyuan Biotechnology Co., Ltd. (Shanghai, China). Anhydrous ethanol was purchased from Beijing Solaibo Technology Co., Ltd. (Beijing, China). Methanol and acetonitrile were purchased from Shanghai Jingpure Biochemical Technology Co., Ltd. (Shanghai, China). Formic acid was purchased from Shanghai Aladdin Biochemical Technology Co., Ltd. (Shanghai, China).

### 2.2. Standard Curve Plotting

A total of 2 mg of rutin was accurately weighed and dissolved in 80% ethanol (50 mL) to prepare a rutin standard solution with the concentration of 0.4 mg/mL. Then, 1 mL of 5% NaNO_2_ solution, 1 mL of 10% Al(NO_3_)_3_ solution, and 10 mL of 10% NaOH solution were added and mixed well in 80% ethanol solution, which reacted for 15 min. The absorbance was measured at 510 nm three times in parallel [14,15]. The standard curve of the rutin solution was plotted with the absorbance value as the vertical coordinate (y) and the mass concentration of rutin as the horizontal coordinate (x, mg/mL), and the regression equation was fitted as y = 11.798x + 0.001 (R^2^ = 0.9997). In this experiment, the NaNO_2_-Al(NO_3_)_3_-NaOH colorimetric method was still used to determine the flavonoid content of *H. spectabile* leaves and to calculate the extraction rate [16].

(1) $$Y\left(\%\right)=\frac{V\times C\times D}{m}\times 100$$

where *Y* is the total flavonoid extraction rate for *H. spectabile* leaves (%), *V* is the total volume of the sample solution (mL), *C* is the concentration of flavonoids (mg/mL), *D* is the number of dilutions, and m is the mass of the sample weighed (g).

### 2.3. Single-Factor Test for Total Flavonoid Extraction

The effects of liquid-to-material ratio (10:1, 15:1, 20:1, 25:1, and 30:1 mL/g), sonication time (20, 40, 60, 80, and 100 min), ethanol concentration (25, 30, 35, 40, and 45%), composite enzyme dosage (0.5, 1.0, 1.5, 2.0, and 2.5%), and enzymatic digestion time (10, 25, 40, 55, and 70 min) on the yield of flavonoids extracted from *H. spectabile* were examined [17,18,19].

### 2.4. Optimization of Extraction Conditions for Total Flavonoid Compounds via Response Surface Analysis

Based on the single-factor test, the liquid-to-material ratio (A), ethanol concentration (B), sonication time (C), and enzyme dosage (D) were further investigated (as the enzymatic digestion time did not affect the extraction rate of total flavonoid compound extraction from *H. spectabile*, therefore, enzymatic digestion time was not selected for analysis), Using Design-Expert V8.0.6.1 software, the extraction conditions of total flavonoid compounds from *H. spectabile* were optimized using four factors and three levels of response surface analysis [9,20,21]. The factor levels were arranged as shown in Table 1.

### 2.5. Isolation and Purification of Flavonoids and Structure Identification for H. spectabile

#### 2.5.1. Selection of Resin Types

In order to extract the total flavonoids from the leaves of *H. spectabile* using the optimal extraction procedure obtained above, the macroporous resin was soaked in 95% ethanol for 24 h and washed repeatedly with distilled water until it was free of alcoholic odor. It was then soaked in 4% NaOH for 2 h and washed until the pH was neutral, followed by soaking in 4% HCl for 2 h and washing until neutral, and finally placed in distilled water for soaking [22,23,24].

According to the resin screening method used in the reference, the static adsorption rate and desorption rate for total flavonoids were used as evaluation indexes. The adsorption and desorption of the total flavonoid crude extract were tested in relation to the resin static adsorption and desorption. The adsorption and desorption effects of eight types of macroporous resins with different polarities, namely, AB-8, D101, NKA-9, HPD-826, HPD-826, HPD-600, S-8, and ADS-17, were screened to obtain the optimal adsorption resins [25,26,27,28]. The physical parameters of the different macroporous resins are shown in Table 2.

#### 2.5.2. Optimum Macroporous Resin Kinetic Curve Experiment

A total of 6 g of macroporous resin was weighed in a 100 mL conical flask, and 25 mL of crude extract of flavonoids from *H. spectabile* leaves was added. The mixture was shaken at 180 rpm and 27 °C for 3 h, then extracted and filtered. A total of 2 mL of the filtrate was taken to measure the A.V. The adsorbed macroporous resin was placed in a 100 mL conical flask, and 25 mL of 80% ethanol was added. Afte shaking and desorbing, 2 mL of the desorbent was taken to measure the A.V. The desorption rate was then calculated [29,30,31,32].

#### 2.5.3. Purification of Total Flavonoids from *H. spectabile* Macroporous Resin—Single-Factor Experiment

Sample volume (2, 4, 6, 8, and 10 mL), sample flow rate (0.5, 1.0, 1.5, 2.0, and 2.5 mL/min), pH (4, 5, 6, 7, and 8), sample concentration (0.4, 0.6, 0.8, 1.0, and 1.2 mg/mL), elution ethanol concentration (40%, 50%, 60%, 70%, 80%, 90%, and 100%), and effects of volume (0, 1, 2, 3, 4, and 5 mL) on the dynamic desorption rate for total flavonoid extract from *H. spectabile* purified on AB-8 macroporous resin were investigated [33,34,35,36].

### 2.6. HPLC-MS Analysis of AB-8 Macroporous Resin Purification

#### 2.6.1. Test Material

The samples were thawed in the refrigerator at 4 °C, and 0.50 mL of the samples was placed in a tube. Then, 0.80 mL of 80% methanol–water was added, fully shocked for 1 min, ultrasonicated at 4 °C for 30 min, left to stand at 4 °C for 60 min, and centrifuged for 10 min. The supernatant was collected for use. The supernatant was concentrated in the concentrator until dry, then 0.50 mL of 80% methanol–water (containing 50 ng/mL internal standard) was added for redissolution, and the mixture was shaken for 5 min. After 60 min at 4 °C, it was centrifuged for 10 min, and the supernatant was collected for testing. Sample A is the extracted liquid obtained by plant dry powder + ethanol + ultrasound + enzyme + concentration. On the basis of sample A, sample B is adsorbed, analyzed, and concentrated with macroporous resin to obtain the extraction solution. Sample C is the extraction solution based on Sample A, which was not adsorbed by macroporous resin. Based on Sample B, Sample D was obtained from the ethyl acetate phase, and Sample E was obtained from the n-butanol phase, separated by a separation funnel [37,38,39,40].

#### 2.6.2. Chromatographic Conditions

Chromatography was performed using an Acquity UPLC HSS T3 column (1.8 µm, 2.1 mm × 100 mm). The separation conditions were as follows: the column temperature was 40 °C, and the flow rate was 0.300 mL/min. A—water (0.1% formic acid) and B—acetonitrile were used as mobile phase for gradient elution. Run time: 18 min, sample size: 6 µL. The sample gradient-elution procedure is shown in Table 3 [22,41,42,43,44,45,46].

#### 2.6.3. Mass Spectrum Condition

The ion source used was an ESI ion source. Curtain gas: 35 arb; collision gas: 9 arb; ion spray voltage: 4000 V; ion source temperature: 400 °C; ion source gas (Ion Source Gas1): 55 arb; and ion source gas (Ion Source Gas2): 55 arb [37].

### 2.7. Analysis of Antioxidant Activity

#### 2.7.1. Preparation of the Sample Solution after Purification and Extraction

The total flavonoids of the crude extracts from *H. spectabile* were purified utilizing macroporous resin, and Vc and rutin were lyophilized and dissolved in absolute ethanol to prepare solutions with concentrations of 0.02, 0.04, 0.06, 0.08, 0.10, 0.12, 0.14, 0.16, 0.18, and 0.20 mg/mL, respectively [46].

#### 2.7.2. Total Reducing Capacity Assay

A total of 2 mL of the purified extract and crude extract of *H. spectabile* with different concentrations was measured in the test tube. A 2.5 mL of 1% potassium ferricyanide solution and 2.5 mL of 0.2 mol/L phosphate buffer (pH = 6.6) were added and mixed very well. The mixture was placed in a water bath at 50 °C for 20 min, cooled, and then 2.5 mL of 10% trichloroacetic acid solution (TCA) was added and mixed well. Centrifugation process was then performed at 4000 r/min for 10 min, 5 mL of supernatant solution in the test tube was taken, 4 mL of pure water was added, and 1 mL of 0.1% of ferric chloride solution. The mixture was mixed well, reacted at room temperature for 15 min, and the absorbance value A was measured at 700 nm with pure water as the blank. Each group was measured 3 times in parallel, and the average value was taken. The larger the absorbance value, the stronger the reducing ability. V_C_ and rutin were used as controls [47].

#### 2.7.3. DPPH Radical-Scavenging Activity Assay

The *DPPH* method was used for determination. A total of 2 mL of *DPPH* solution of 0.2 mmol/L was taken, and 2 mL of sample solution was added, shaken well, and left to stand in the dark for 30 min. Absolute ethanol was used as the reference solution, and the absorbance value *A*_1_ was measured at *A*_517_. At the same time, the absorbance value *A*_0_ of a mixture 2 mL *DPPH* solution and an equal volume of absolute ethanol mixture was determined, as well as the absorbance value *A*_2_ of a mixture of ethanol solution and an equal volume of absolute ethanol containing different concentrations of total flavonoids. The above experiments were repeated with *Vc* and rutin ethanol solution with the same concentration gradient as the control. The scavenging rate of *DPPH* free radicals by each sample solution was calculated according to the following formula [48,49].

(2) $$Scavenging\;rate\;of\;DPPH\;radicals\left(\%\right)=\frac{A_{0}-\left(A_{1}-A_{2}\right)}{A_{0}}\times 100\%$$

#### 2.7.4. Hydroxyl Radical-Scavenging Ability Assay

The salicylic acid method was used to determine antioxidant activity. A total of 1 mL of 2 mmol/L FeSO_4_ solution and 1 mL of 6 mmol/L H_2_O_2_ solution were added to a 10 mL colorimetric tube and shaken well. Then, 1 mL of 6 mmol/L salicylic acid–ethanol solution and 1 mL of sample solution of different concentrations were added. The solution was diluted to volume with distilled water and placed in a 37 °C water bath for 15 min. Distilled water was used as control, and the absorbance was measured at 510 nm, recorded as *A_i_*. The absorbance measured by taking 1 mL of distilled water instead of H_2_O_2_ solution was recorded as *A_j_*. The absorbance measured by using 1 mL of distilled water instead of the sample solution was recorded as *Ac*. The measurements were performed in parallel triplicate. The above experiments were repeated with *V_C_* and rutin ethanol solutions with the same concentration gradient as the control [50].

(3) $$Scavenging\;rate\;of\cdot OH\;radicals\left(\%\right)=\frac{A_{c}-\left(A_{i}-A_{j}\right)}{A_{c}}\times 100\%$$

#### 2.7.5. ABTS^+^ Radical-Scavenging Ability Assay

Firstly, 7 mM of ABTS solution and 2.5 mM of potassium persulfate solution were prepared and mixed in equal volume, then left to stand at room temperature for 14 h, protected from light, to obtain the ABTS reserve solution. Before use, the ABTS reserve solution was diluted with 75% ethanol to make the working solution, with an absorbance value of 0.7 ± 0.02 at 734 nm. Then, 0.2 mL of the total flavonoid extract of *H. spectabile* was added to 5.8 mL of the working solution of ABTS with the gradient concentration of 50, 100, 150, 200, 250, and 300 µg·mL^−1^. Then, the reaction was carried out for 10 min under the light. Finally, the absorbance values were measured at 734 nm, and the clearance was calculated. Anhydrous ethanol was used as a blank control, and the same mass concentration of L-ascorbic acid was used as a positive control, where *A*_1_ is the absorbance value of the blank group, *A*_2_ is the absorbance value of the total flavonoid group of *H. spectabile*, and *A*_3_ is the absorbance value of the flavonoid background group [51].

(4) $$Scavenging\;rate\;of\;{ABTS}^{-+}\;radicals(\%)=\frac{A_{1}-\left(A_{2}-A_{3}\right)}{A_{1}}\times 100$$

### 2.8. Method of Analysis

SAS 9.2 software was used to statistically analyze the obtained data, and the test results were expressed as the mean ± standard deviation (mean ± SD). Duncan’s analysis was used for multiple comparisons, and *p* < 0.05 was considered to indicate a significant difference.

## 3. Results and Discussion

### 3.1. Single-Factor Test Results for the Extraction of Total Flavonoids from H. spectabile

#### 3.1.1. Effect of Liquid–Material Ratio on the Extraction Rate of Total Flavonoids from *H. spectabile*

**Figure 1:**
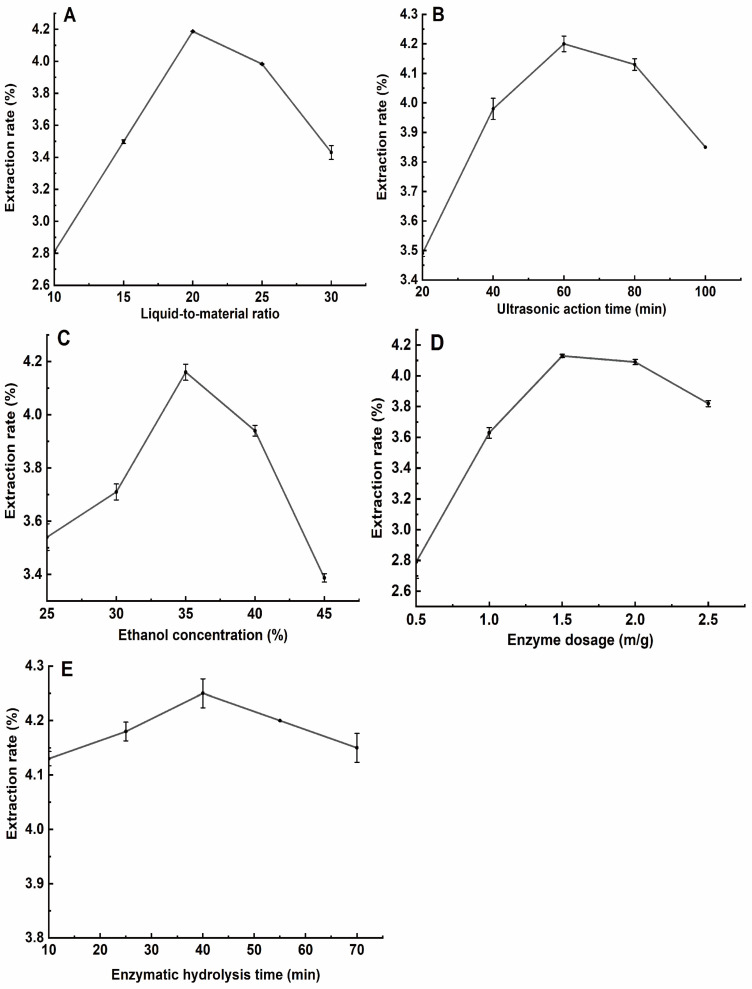
Effects of the liquid-to-material ratio (**A**), ultrasonic action time (**B**), ethanol concentration (**C**), enzyme dosage (**D**), and enzymatic hydrolysis time (**E**) on the rate of total flavonoid compound extraction from the leaves of *H. spectabile*.

For the leaves of *H. spectabile* (Figure 1A), the extraction rate of flavonoids was first increased and then decreased with the increase in the liquid-to-material ratio. As the liquid-to-material ratio was increased from 10:1 to 20:1, the total flavonoid yield was increased and reached a peak at 20:1 with an extraction rate of 4.187%. With the continuous improvement in the liquid-to-material ratio, the internal and external osmotic pressure tended to be stable, and the mass transfer and diffusion of flavonoids also tended to be stable [52]. The extraction rate of flavonoids increased slowly. Considering that increasing the solvent could lead to resource waste, a liquid-to-material ratio of 15:1–25:1 mL/g was selected for subsequent optimization experiments.

#### 3.1.2. Effect of Ultrasonic Extraction Time on the Rate of Total Flavonoid Extraction from *H. spectabile*

For *H. spectabile* leaves (Figure 1B), as the extraction time increased from 20 to 60 min, the total flavonoid yield increased and reached a peak at 60 min, with an extraction rate of 4.2%. As the extraction time continued to increase, the flavonoid extraction rate decreased, which may be due to the fact that as the ultrasound time increased, the temperature of the ultrasound machine also increased, thereby destroying the biological activity of the total flavonoids of *H. spectabile* [53]. Therefore, a microwave extraction time in the range of 40–80 min was chosen for the following tests.

#### 3.1.3. Effect of Ethanol Volume Fraction on the Rate of Total Flavonoid Extraction from *H. spectabile*

The extraction rate of total flavonoids from *H. spectabile* leaves (Figure 1C) gradually increased with ethanol volume fractions between 25 and 35%, peaking at 35% with an extraction rate of 4.16%. As the ethanol volume fraction continued to increase, the extraction rate of total flavonoids gradually decreased. Increasing the permeability of the solution to the material could enhance the extraction rate of flavonoids, but as the ethanol volume fraction increased, the biological activity of the flavonoids was destroyed. This was due to a reduction in water molecules, which inhibited the dissolution of flavonoids and thereby reduced the extraction rate [54]. Therefore, 30–40% ethanol (by volume) was selected for subsequent optimization testing.

#### 3.1.4. Effects of the Amount of Complex Enzyme on the Rate of Total Flavonoid Extraction from *H. spectabile*

In the case of *H. spectabile* leaves (Figure 1D), the flavonoid extraction rate gradually increased as the concentration of complex enzyme was increased from 0.5 and 1.5%. When the amount of complex enzyme was 1.5%, the maximum extraction yield of flavonoids was 4.13%. For this reason, a complex enzyme dosage of 1.0–2.0% was selected for further testing.

#### 3.1.5. Effect of Enzymolysis Time on the Rate of Total Flavonoid Extraction from *H. spectabile*

The effects of different enzymatic hydrolysis times on the rates of flavonoid extraction from leaves of *H. spectabile* are shown in Figure 1E. As the enzymatic hydrolysis time increased, the yield of total flavonoids first increased and then decreased. However, the effect of the enzymatic hydrolysis time on the total flavonoid extraction rate was not significant. When the enzymatic hydrolysis time exceeded 40 min, the yield decreased slightly. This may be because, with longer enzymatic hydrolysis times, the efficiency of the hydrolysis did not improve further. Also, some of the heat-resistant flavonoids (flavonosides) in the dissolved total flavonoids may be decomposed so that when the enzymatic hydrolysis time exceeds 40 min, the total flavonoids yield slowly decreases [55]. Therefore, the enzymatic hydrolysis time of 40 min was chosen.

### 3.2. Optimization Results for a Response Surface Experiment for the Extraction of Total Flavonoids from H. spectabile

#### 3.2.1. Establishment and Significance Test of the Response Mode

Based on the results of the single-factor test, the solid–liquid ratio, complex enzyme dosage, ethanol concentration, and ultrasonic time were selected as independent variables, with the flavonoid extraction rate (Y) as the response variable. A Box–Benhnken experiment was conducted to design the extraction parameters for the roots, stems, leaves, and flowers of *H. spectabile* using Design Expert 8.6.1. Each test had 29 test points, including 24 factorial points and 5 central points. The central test was repeated five times to estimate the pure test error [56]. Table 4 shows the response surface design and results, while Table 5 shows the analysis of variance (ANOVA).

As for *H. spectabile* leaves, regression fitting analysis was carried out, and the results are shown in Table 5. The quadratic multiple regression equation model was finally obtained as follows:

Y = 4.17 + 0.12A + 0.17B + 0.17C + 0.19D + 0.088AB + 0.065AC + 0.14AD + 0.13BC + 0.063BD + 0.068CD − 0.40A^2^ − 0.38B^2^ − 0.38C^2^ − 0.38D^2^ (5)

According to the analysis of variance (Table 5), the factors ranked as follows in terms of the degree to which they influenced the flavonoid extraction rate in microwave-assisted extraction, based on the F value: D (amount of complex enzyme) > B (ethanol concentration) > C (ultrasonic time) > A (solid–liquid ratio). The overall model equation (P-value) shown in the table was significant when simulated < 0.0001, and the difference between the different treatment conditions was extremely significant: (P) _mismatch_ = 0.2333 > 0.05 indicated that the difference was not significant. The R^2^ coefficient was 0.9815; this is higher than 0.9, indicating that the model had a good degree of fit.

#### 3.2.2. Analyzing Response Surface Maps and Contour Maps

**Figure 2:**
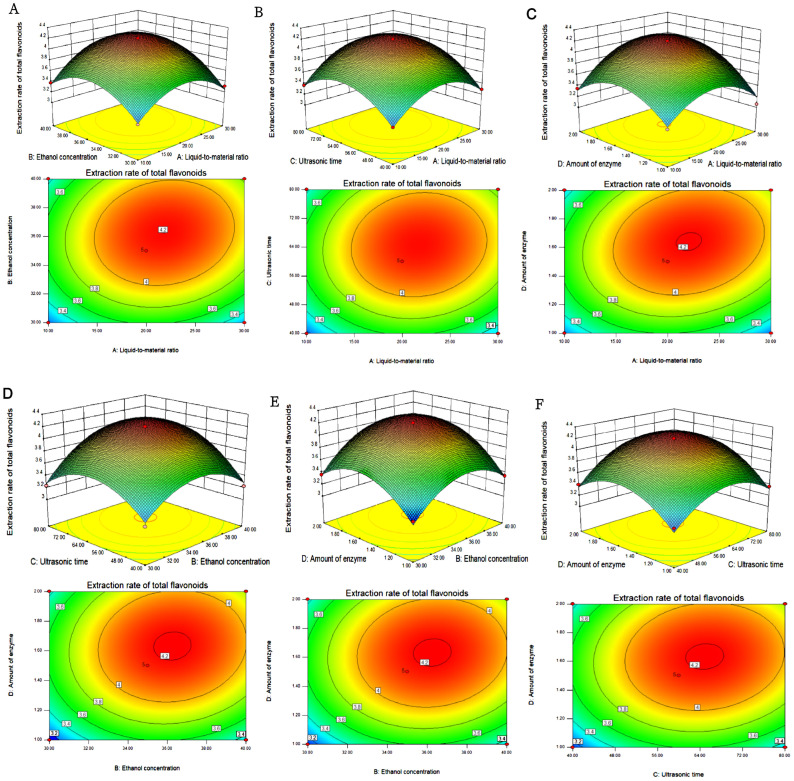
Response surface plots for the pairwise effects on the extraction yield of total flavonoids from *H. spectabile* leaves: (**A**) liquid–solid ratio and ethanol concentration; (**B**) liquid-to-material ratio and ultrasonic time; (**C**) liquid-to-material ratio and enzyme dosage; (**D**) ultrasound time and ethanol concentration; (**E**) the amount of enzyme and concentration of ethanol; (**F**) enzyme dosage and ultrasound time.

Based on the results shown in Table 4 and Table 5 and Figure 2, the optimum process conditions for extracting total flavonoids from *H. spectabile* can be deduced. Total flavonoid extraction was 4.23% at a 20.98:1 mL/g liquid–solid ratio, 35% ethanol concentration, 60 min ultrasonication time, and 1.5% total amount of bis-enzyme. With the limitation of operating conditions, a 20:1 mL/g liquid–solid ratio, 35% ethanol concentration, 60 min sonication time, and 1.5% total amount of dual enzyme were the optimality extraction conditions. Under these conditions, the extraction level of total flavonoids was 4.22% after three repeated iterations [57]. The above results indicate that the parameters for the dual-enzyme-assisted ultrasonication extraction process were accurate and reliable when using the response surface analysis method; therefore, they could be implemented.

### 3.3. Separation and Purification of Total Flavonoids from H. spectabile

#### 3.3.1. Screening of Macroporous Resins

In this experiment, eight types of macroporous resins with different polarities were selected for static adsorption and desorption (Table 6). According to static adsorption and analytical tests, the adsorption of total flavonoids of *H. spectabile* by AB-8 macroporous resin was as high as 81.70%, which was higher than that of the other seven macroporous resins. The static desorption rate was lower than that of D101, D101, and X-5 macroporous resins, up to 85.35%, and the desorption effect was better, but the adsorption rates of D101, ADS-171, and X-5 macroporous resins for total flavonoids were 60.63%, 58.36%, and 68.52%, respectively. The adsorption capacity was worse than that of AB-8 macroporous resin. By comparing the adsorption and desorption rates of 8 macroporous resins in Table 6, AB-8 macroporous resin was selected for the separation and purification of total flavonoids from *H. spectabile* [58].

#### 3.3.2. Static Adsorption and Desorption Kinetic Curves of AB-8

**Figure 3:**
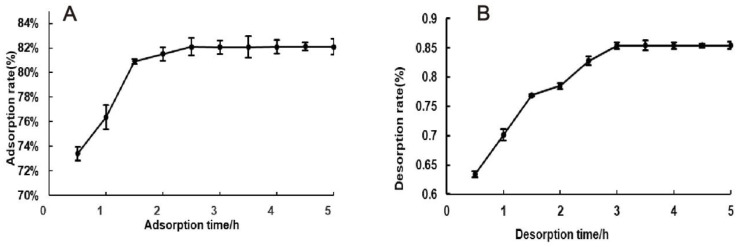
Kinetic adsorption curves. (**A**) Effect of adsorption time on adsorption rate; (**B**) effect of desorption time on desorption rate.

Since the adsorption rate and desorption rate of AB-8 macroporous resin were relatively high, the adsorption and desorption kinetic curves of AB-8 macroporous resin on the total flavonoids in *H. spectabile* solution were investigated, as shown in Figure 3. It can be seen in Figure 3A that the macroporous resin rapidly absorbed a large number of flavonoids within 1 h, with the adsorption rate tending to become saturated and gradually reaching equilibrium after 1 h. Figure 3B shows that the flavonoids were rapidly released within 3 h, and the equilibrium state was reached after 3 h. In summary, the optimum adsorption time for AB-8 macroporous adsorption resin is 1.5 h, and the desorption time is 3 h.

#### 3.3.3. Single-Factor Adsorption Test Results

**Figure 4:**
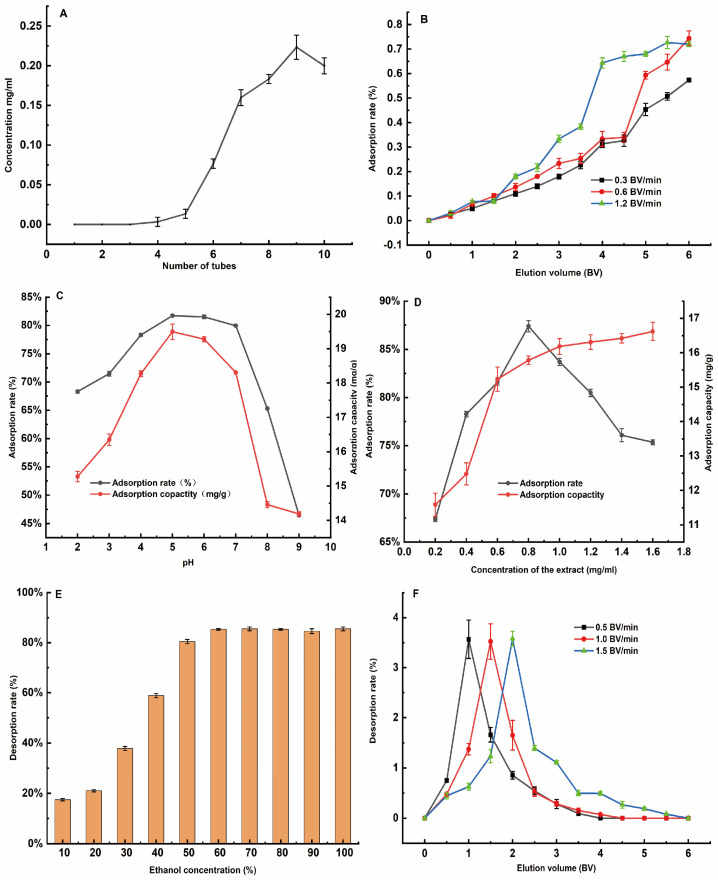
Adsorption and elution single-factor results: (**A**) Leakage curves; (**B**) effect of elution volume on adsorption rate; (**C**) effect of pH on adsorption rate and adsorption; (**D**) effect of extract concentration on adsorption rate and adsorption; (**E**) effect of ethanol concentration on desorption rate; (**F**) effect of elution volume on desorption rate.

As shown in Figure 4A, leakage occurred when the 8th tube was collected, and the leakage showed a steady trend when the 10th tube was collected. The results show that the maximum amount of liquid added to the sample was approximately two times the volume of the column.

As the liquid volume increased, the more flavonoids that were not adsorbed by the macroporous resin, the weaker the adsorption capacity of the resin (Figure 4B). When the liquid volume was less than 3.5 BV, the adsorption rate increased slowly, while when the loading reached 4.5 BV, the adsorption rate increased obviously. Therefore, the optimum loading flow rate was 1.2 BV/min, and the maximum loading volume was 4.5 BV.

As the pH of the solution increased, the adsorption rate increased slowly and strongly (Figure 4C). When the pH value was 5, the adsorption rate reached the maximum value, and the maximum adsorption rate was 76.12%. As the pH continued to increase, the adsorption rate did not change significantly, but when the pH increased to more than 7, the adsorption rate decreased sharply. Therefore, 5 was chosen as the pH of the initial solution to obtain the best flavonoid adsorption capacity.

As shown in Figure 4D, the adsorption rate of the macroporous resin increased significantly with the increase in the extraction solution concentration. When the extraction solution concentration was greater than 0.8 mg/mL, the adsorption rate decreased rapidly, showing a trend of first increasing and then decreasing. When the inflection point of the adsorption rate curve was around 0.8 mg/mL, the adsorption rate reached the maximum, and the maximum value was 87.46%. Therefore, 0.8 mg/mL was selected as the optimum loading solution concentration.

#### 3.3.4. Results of the Elution Single-Factor Test

Figure 4E shows that, in principle, 60% ethanol can elute most of the adsorbed flavonoids from AB-8 resin. Therefore, 50 or 60% ethanol can be selected as the eluent. As shown in Figure 4F, a peak began to appear from the fourth tube, and from the tenth tube, the concentration of flavonoids in the eluent peaked and then decreased. After 30 mL, the flavonoids adsorbed on AB-8 had essentially been eluted. Thus, 2 BV was more appropriate to choose as the eluent volume.

### 3.4. Identifying the Composition of the Total Flavonoids of H. spectabile

#### 3.4.1. HPLC-MS Identification and Analysis of Flavonoids

*H. spectabile* contains a wide variety of flavonoids, the six most important of which are fisetin, myricitrin, luteolin, rutin, kaempferol, and phloretin. The flavonoid contents of the five groups of *H. spectabile* samples were determined using HPLC-MS. The results show that HPLC chromatograms with good separation could be obtained for samples from each *H. spectabile* treatment group. The peak times corresponding to fisetin, myricitrin, luteolin, rutin, kaempferol, and phloretin were 6.51, 8.74, 8.92, 8.01, 8.28, and 9.77 min, respectively, and the peak time of the samples from all the treatment groups remained consistent. The results indicate that the purification of the top six flavonoids using macroporous resin and subsequent extraction and concentration did not change the peak time of the samples, and the types of flavonoids remained the same.

**Figure 5:**
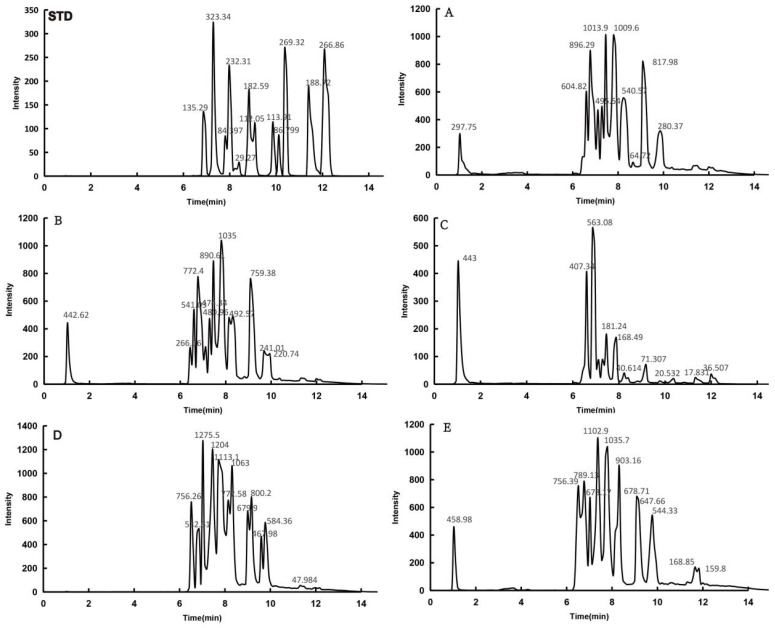
Liquid chromatogram. STD is the standard spectrum of flavonoids. (**A**) Liquid chromatogram of extract concentration of *H. spectabile* sample; (**B**) liquid chromatogram of concentrate after purification of *H. spectabile* sample; (**C**) liquid chromatogram of filtrate after the adsorption of *H. spectabile* sample; (**D**) liquid chromatogram of ethyl acetate phase of *H. spectabile* sample; (**E**) liquid chromatogram of the n-butanol phase of the *H. spectabile* sample.

The HPLC results for the five groups of samples are shown in Figure 5. The flavonoids of *H. spectabile* were well isolated and could be used for subsequent identification via mass spectrometry. The molecular ion peaks obtained via primary mass spectrometry were used to determine the possible molecular weight of the substance. Based on the fragment ion peak obtained via secondary mass spectrometry, the structure of the unknown compound was deduced by referring to the bond-breaking modes of different compounds and related literature reports.

#### 3.4.2. Flavonoid Content Analysis

To further analyze the effects of the extraction methods, the macroporous resin adsorption, extraction time, and concentration of the flavonoid content in the five groups of samples, as well as the peak time, peak area, and flavonoid content in each sample, were explored. The results are shown in Table 7. The peak time of the five groups of samples is the same, and the flavonoid content is different. The species are basically the same. The results show that the extraction method, macroporous resin adsorption, extraction time, and concentration had no effect on the flavonoid components of the sample but affected the content of each flavonoid.

In the follow-up test, mass spectrometry and liquid chromatography were combined to analyze and identify flavonoids in five groups of samples, and the results are shown in Table 8. The HPLC-MS method was used to determine the content of flavonoids in *H. spectabile*, and 46 types of flavonoids were detected in the sample. Among them, the flavonoids with higher contents were fisetin, myricetin, luteolin, rutin, kaempferide, and phloretin. Ethanol extraction, macroporous resin adsorption, extraction time, and concentration affected the content of flavonoids in the sample. However, the peak time and types of each component were not affected, which proved that the HPLC-MS detection method could be effectively used to analyze and identify *H. spectabile* samples.

### 3.5. Study of the Antioxidant Activity of the Flavonoids in H. spectabile

#### 3.5.1. Total Reducing Capacity of *H. spectabile* Flavonoid Extract

**Figure 6:**
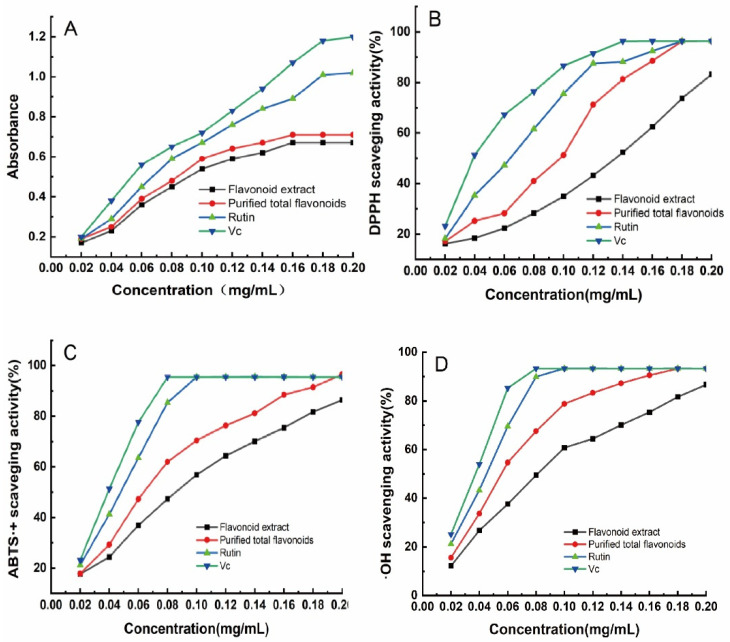
Study of the antioxidant activity of the flavonoids in *H. spectabile*. Determination of (**A**) total reducing capacity, (**B**) DPPH free radical-scavenging capacity, (**C**) ABTS^·+^ free radical-scavenging capacity, and (**D**) ·OH radical-scavenging capacity.

Flavonoids are polyhydroxyl compounds that can provide hydrogen atoms to reduce potassium ferricyanide and show strong antioxidant capacity [58,59]. The total reducing power of the sample can reflect antioxidant performance. Based on the above results, the antioxidant performance of *H. spectabile* was investigated. As can be seen in Figure 6A, when the sample concentration was in the range of 0.02–0.18 mg/mL, the concentration of the flavonoid extract showed a good linear relationship with the reducing power; the concentration of flavonoid extract increased before and after purification, and the reducing power increased. When the concentration of the flavonoid extract was 0.16 mg/mL, the absorbance values of the purified flavonol and unpurified flavonol were basically unchanged. In this concentration range, the reducing power of the purified flavonol was always higher than that of the unpurified flavonol. The above results indicate that the total flavonoids of *H. spectabile* had antioxidant capacity, and the reducing power reached saturation at a concentration of 0.16 mg/mL.

#### 3.5.2. DPPH Free Radical-Scavenging Ability of *H. spectabile* Flavonoid Extract

By measuring the DPPH free radical-scavenging rates, the antioxidant activities of *H. spectabile* flavonoid extract and extract subjected to AB-8 macroporous resin purification were compared. The results are shown in Figure 6B. In the concentration range of 0.02–0.18 mg/mL, the total flavonoids and purified flavonoids of *H. spectabile* showed varying degrees of free radical-scavenging ability, but the scavenging effect for DPPH free radicals was significant and concentration-dependent [60]. At the same concentration, the free radical-scavenging ability of purified total flavonoids was higher than that of unpurified total flavonoids, indicating a correlation between the level of antioxidant and flavanol content. The components ranked as follows in terms of DPPH release: Vc > rutin > purified total flavonoids > crude extract of total flavonoids. When the mass concentration was 0.14 mg/mL, the vitamin C clearance rate reached 96.32%, the rutin clearance rate reached 88.13%, the clearance rate for flavonoids purified using AB-8 macroporous resin was 81.25%, and the unpurified flavonoid clearance rate was 52.33%, showing strong free radical-scavenging ability. This is mainly related to the flavonoid content. After purification, the flavonoid content in the flavonoid extract was increased, and the free radical-scavenging effect was improved. The results indicate that the total flavonoids of *H. spectabile* had a good scavenging effect on DPPH free radicals.

#### 3.5.3. ABTS^·+^ Free Radical-Scavenging Ability of *H. spectabile* Flavonoid Extract

As can be seen in Figure 6C, within the mass concentration range designed in this experiment, the scavenging ability of the flavonoid extract and extract purified with AB-8 macroporous resin for ABTS^·+^ free radicals gradually increased with an increasing concentration. The specimens ranked as follows in terms of antioxidant capacity: *Vc* > rutin > purified total flavonoids > crude extract of total flavonoids. At 0.10 mg/mL, the ABTS^·+^ free radical-scavenging rate of the extract purified with AB-8 macroporous resin was 70.41%, while that of the flavonoid extract was 56.78%. Compared with the flavonoid extract, the extract purified with AB-8 macroporous resin had better scavenging ability for ABTS^·+^ free radicals. This may have been due to the improved purity following AB-8 macroporous resin purification, which removed impurities that had no or less antioxidant activity, thus improving the antioxidant capacity [59,60]. In conclusion, the extract subjected to AB-8 macroporous resin purification showed excellent scavenging in terms of DPPH· and ABTS^·+^ free radicals, mainly due to the flavonoids’ structure-rich phenolic hydroxyl structures, which contribute to the scavenging of free radicals.

#### 3.5.4. ·OH Free Radical-Scavenging Ability of *H. spectabile* Flavonoid Extract

·OH free radicals are a type of active oxygen free radicals. As shown in Figure 6D, the scavenging ability of the flavonoid extract and the extract subjected to AB-8 macroporous resin purification for ·OH free radicals gradually increased with an increasing concentration. When the concentration of the extract purified with AB-8 macroporous resin increased from 0.02 mg/mL to 0.2 mg/mL, the clearance rate increased from 15.57% to 93.26%, indicating that the scavenging ability of total flavonoids for hydroxy free radicals showed a good dose–response relationship with the concentration of the sample. When the concentration of total flavonoids was greater than 0.06 mg/mL, the scavenging rate for hydroxyl free radicals was greater than 50%, so this extract could be used as an antioxidant to remove hydroxyl free radicals.

## 4. Conclusions

In this study, the extraction of total flavonoids from the leaves of *H. spectabile* was optimized using a complex enzyme-assisted ultrasonic method through a single-factor test, response surface analysis, and a quadratic regression equation model. To establish the separation and purification as well as increase the total flavonoids extracted from *H. spectabile*, the AB-8 macroporous resin with a higher adsorption rate for total flavonoids was selected. The total flavonoid content of the *H. spectabile* samples was effectively analyzed and identified via HPLC-MS. According to the oxygen free radical-scavenging test and redox test in vitro, the *H. spectabile* flavonoid extract had a strong antioxidant capacity, which was positively correlated with the *H. spectabile* flavonoid concentration; the *H. spectabile* flavonoids were more sensitive to DPPH, and the required concentration was lower. Compared to the original flavonoid extract, the main advantage of the purified flavonoid extract was that it had strong antioxidant activity at very low concentrations. This study provides theoretical and technical support for the further development and utilization of total flavonoids from *H. spectabile*.

## Figures and Tables

**Table 1:** Response surface test factors and levels.

Factors	Levels		
	−1	0	1
A Liquid-to-material ratio (mL/g)	15:1	20:1	25:1
B Ethanol concentration (%)	30	35	40
C Ultrasonic extraction time (min)	40	60	80
D Amount of enzyme (%)	1.0	1.5	2

**Table 2:** Physical parameters of different macroporous resins.

Macroporous Resin	Polarity	Surface Area (m^2^/g)	Pore Size (nm)
AB-8	Weak polarity	450–530	13.0–14.0
D101	Non-polar	600–700	10.0–12.0
S-8	Polarity	90–150	25.0–30.0
HPD-600	Polarity	480–520	8.0
NKA-9	Polarity	170–250	15.5–16.5
HPD-826	Polarity	500–600	9.0–10.0
X-5	Non-polar	500–550	29.0–30.0
ADS-17	Non-polar	90–150	25.0–30.0

**Table 3:** Sample gradient-elution procedure.

Time	Flow Rate	%B
Initial	0.300	10.0
1.00	0.300	10.0
14.00	0.300	90.0
15.00	0.300	90.0
15.10	0.300	10.0
18.00	0.300	10.0

**Table 4:** Box–Behnken test and results of *H. spectabile* leaves.

Experiment Number	A	B	C	D	Total Flavonoid Extraction Rate Y (%)
1	−1	−1	0	0	3.166
2	−1	0	1	0	3.369
3	0	0	0	0	4.109
4	0	1	−1	0	3.226
5	−1	0	−1	0	3.169
6	1	1	0	0	3.88
7	0	−1	−1	0	3.136
8	1	0	1	0	3.76
9	−1	0	0	−1	3.18
10	0	−1	1	0	3.236
11	0	1	0	−1	3.345
12	0	1	0	1	3.796
13	0	−1	0	1	3.367
14	0	0	0	0	4.207
15	0	0	−1	−1	3.188
16	0	0	−1	1	3.399
17	0	0	0	0	4.206
18	−1	0	0	1	3.35
19	0	0	0	0	4.206
20	0	0	1	−1	3.365
21	0	1	1	0	3.838
22	1	0	0	1	3.775
23	1	−1	0	0	3.316
24	0	0	0	0	4.108
25	−1	1	0	0	3.378
26	0	−1	0	−1	3.167
27	0	0	1	1	3.846
28	1	0	0	−1	3.358
29	1	0	−1	0	3.298

**Table 5:** Variance analysis of regression model of *H. spectabile* leaf response surface test.

Source of Variance	Sum of Squares	Degrees of Freedom	Mean Squared	F-Number	*p*	Significance
Model	3.94	14	0.28	52.94	<0.0001	***
A	0.18	1	0.18	34.06	<0.0001	***
B	0.36	1	0.36	67.41	<0.0001	***
C	0.33	1	0.33	62.50	<0.0001	***
D	0.41	1	0.41	77.86	<0.0001	***
AB	0.03	1	0.03	5.82	0.0301	*
AC	0.02	1	0.02	3.22	0.0942	*
AD	0.07	1	0.07	14.05	0.0022	*
BC	0.07	1	0.07	12.31	0.0035	*
BD	0.02	1	0.02	2.96	0.1074	*
CD	0.02	1	0.02	3.42	0.0855	*
A2	1.02	1	1.02	191.65	<0.0001	***
B2	0.92	1	0.92	173.72	<0.0001	***
C2	0.94	1	0.94	176.15	<0.0001	***
D2	0.93	1	0.93	175.45	<0.0001	***
Residual error	0.075	14	5.323 × 10^−3^			
Missing fit	0.063	10	6.303 × 10^−3^	2.19	0.2333	
Pure error	0.011	4	2.872 × 10^−3^			
Total deviation	4.02	28				
		R2	0.9815			
		R2Adj	0.9629			

Note: *: significant difference (*p* < 0.05); ***: extremely significant difference (*p* < 0.01).

**Table 6:** Adsorption/desorption capacities and desorption ratios for flavonoids on different resins.

Resin Type	Adsorption Rate (%)	Desorption Rate (%)
ADS-17	58.36%	89.72%
D101	60.63%	97.67%
X-5	68.52%	89.55%
S-8	75.46%	84.97%
HPD-600	77.14%	83.02%
HPD-826	77.55%	83.98%
NKA-9	78.22%	80.23%
AB-8	81.70%	85.35%

**Table 7:** Contents of flavonoids in (**A**) sample A (extract concentration of *H. spectabile*), (**B**) sample B (concentration after purification of *H. spectabile* sample), (**C**) sample C (concentration after purification of *H. spectabile* sample), (**D**) sample D (concentration after purification of *H. spectabile* sample), and (**E**) sample E (concentration after purification of *H. spectabile* sample).

**(A)**				
**Number**	**Substance Name**	**Peak Time (min)**	**Peak Area** **(*m*/*z*)**	**Content (ng/mL)**
1	Fisetin	6.51	8,260,000	33,317.94651
2	Myricitrin	8.74	70,100,000	17,386.46613
3	Phloretin	9.77	23,200,000	11,933.10595
4	Luteolin	8.92	1,670,000	6065.768006
5	Quercitrin	10.2	45,900,000	2907.228018
6	Astragalin	7.88	51,400,000	2774.128377
7	L-Epicatechin	8.48	24,000,000	2547.56823
8	Kaempferol	8.28	1,370,000	2026.947908
9	Neohesperidin	7.99	71,400,000	1604.706504
10	Rutin	8.01	12,200,000	1555.740433
**(B)**				
**Number**	**Substance Name**	**Peak Time (min)**	**Peak Area** **(mAU/min)**	**Content (ng/mL)**
1	Fisetin	6.51	4,160,000	12,042.10526
2	Myricitrin	8.74	38,600,000	6870.550162
3	Luteolin	8.92	1,960,000	5109.004739
4	Phloretin	9.77	12,200,000	4503.355705
5	Quercitrin	10.2	56,200,000	2554.545455
6	Kaempferol	8.28	2,350,000	2495.173745
7	Rutin	8.01	22,500,000	2059.06822
8	Astragalin	7.88	43,600,000	1688.732394
9	L-Epicatechin	8.48	13,700,000	1043.628809
10	Vitexin	7.50	793,000	843.6170213
**(C)**				
**Number**	**Substance Name**	**Peak Time (min)**	**Peak Area** **(mAU/min)**	**Content (ng/mL)**
1	Rutin	8.01	18,600,000	2208.913897
2	Myricitrin	8.74	7,410,000	1711.591195
3	Quercitrin	10.2	7,400,000	436.5024289
4	Neohesperidin	7.99	18,800,000	393.4991381
5	Protocatechuic acid	7.37	19,600,000	338.7242852
6	Hesperidin	8.00	2,030,000	271.3283015
7	Astragalin	7.88	5,120,000	257.3486722
8	Glycitin	11.5	2,880,000	84.59146169
9	Protocatechualdehyde	7.20	1,260,000	82.50577772
10	Tangeretin	12.1	4,490,000	80.31816182
**(D)**				
**Number**	**Substance Name**	**Peak Time (min)**	**Peak Area** **(mAU/min)**	**Content (ng/mL)**
1	Fisetin	6.51	8,030,000	61,360.33017
2	Myricitrin	8.74	76,200,000	35,803.23223
3	Phloretin	9.77	24,800,000	24,165.2424
4	Luteolin	8.92	2,930,000	20,160.9461
5	Kaempferol	8.28	6,140,000	17,209.34555
6	Vitexin	7.50	2,390,000	6711.708735
7	Quercitrin	10.2	55,700,000	6683.370977
8	Astragalin	7.88	56,200,000	5746.107077
9	L-Epicatechin	8.48	25,000,000	5027.22768
10	Rutin	8.01	20,500,000	4952.279327
**(E)**				
**Number**	**Substance Name**	**Peak Time (min)**	**Peak Area** **(mAU/min)**	**Content (ng/mL)**
1	Fisetin	6.51	8,260,000	33,317.94651
2	Myricitrin	8.74	70,100,000	17,386.46613
3	Phloretin	9.77	23,200,000	11,933.10595
4	Luteolin	8.92	1,670,000	6065.768006
5	Quercitrin	10.2	45,900,000	2907.228018
6	Astragalin	7.88	51,400,000	2774.128377
7	L-Epicatechin	8.48	24,000,000	2547.56823
8	Kaempferol	8.28	1,370,000	2026.947908
9	Neohesperidin	7.99	71,400,000	1604.706504
10	Rutin	8.01	12,200,000	1555.740433

**Table 8:** Contents of flavonoid in five groups of samples detected via HPLC-MS (ng/mL).

Sample Name	Fisetin	Myricetin	Luteolin	Rutin	Kaempferide	Phloretin
A	13,965.77	202.60	5046.74	2376.83	455.00	1984.23
B	12,042.11	183.71	5109.00	2059.07	405.91	4503.3
C	67.99	6.52	45.33	2208.91	8.16	14.32
D	61,360.33	1323.14	20,160.95	4952.28	1642.04	24,165.24
E	33,317.95	619.44	6065.77	1555.74	651.31	11,933.11

## Data Availability

The original contributions presented in the study are included in the article; further enquiries can be directed to the corresponding authors.
